# Soluble B7-H5 Is a Novel Diagnostic, Severity, and Prognosis Marker in Acute Pancreatitis

**DOI:** 10.1155/2021/1223850

**Published:** 2021-10-08

**Authors:** Ruoxin Xu, Ju Gong, Wei Chen, Yakang Jin, Jian Huang

**Affiliations:** ^1^Department of Emergency, Internal Medicine of the First Affiliated Hospital, Suzhou University, Suzhou, 215000 Jiangsu, China; ^2^Department of Emergency, Changshu No. 2 People's Hospital, Suzhou, 215500 Jiangsu, China; ^3^College of Pharmaceutical Sciences, Suzhou University, Suzhou, 215000 Jiangsu, China

## Abstract

As an important ligand in T lymphocyte costimulatory pathways, B7-H5 is involved deeply in the immune response in various diseases. However, its clinical usefulness as an early indicator in acute pancreatitis (AP) remains unclear. In this study, the levels of sB7-H5 and cytokines in plasma samples of 75 AP patients, 20 abdominal pain patients without AP, and 20 healthy volunteers were determined. Then, the correlation of sB7-H5 and clinical features, cytokines, the Ranson score, APACHE II score, Marshall score, and BISAP score was analysed, and the value of sB7-H5 for diagnostic, severity, and prognosis of AP was evaluated. We found that the levels of sB7-H5 were specifically upregulated in AP patients. Receiver operating characteristic (ROC) analysis revealed that sB7-H5 can identify AP patients from healthy or abdominal pain patients with 78.9% or 86.4% sensitivity and 93.3% or 90.0% specificity. Further analysis showed that the levels of sB7-H5 were significantly correlated with WBC (*p* = 0.004), GLU (*p* = 0.008), LDH (*p* < 0.001), Ca^2+^ (*p* = 0.006), AST (*p* = 0.009), PLT (*p* = 0.041), IL-6 (*p* < 0.001), IL-10 (*p* < 0.001), and TNF-*α* (*p* < 0.001). And levels of sB7-H5 were gradually increased among patients with mildly acute pancreatitis (MAP), moderately severe acute pancreatitis (MSAP), and severe acute pancreatitis (SAP). It can distinguish the severity of AP with good sensitivity and specificity. Moreover, when dividing the patients into two groups according to the median level of sB7-H5, the local complication and length of stay of low levels of the sB7-H5 group were significantly less than those in high levels of the sB7-H5 group. And the levels of sB7-H5 in AP patients were significantly correlated with the Ranson score (*p* < 0.001), APACHE II score (*p* < 0.001), Marshall score (*p* < 0.001), and BISAP score (*p* < 0.001). The AUCs of assessing local complications of sB7-H5 at day 1 and day 3 were 0.704 (*p* = 0.0024) and 0.727 (*p* = 0.0373). These results showed the potential value of sB7-H5 as a diagnostic, severity, and prognosis marker of AP.

## 1. Introduction

Acute pancreatitis (AP) is a severe inflammation of the pancreas caused by local pancreatic damage, which may lead to multiorgan failure or death. The incidence of acute pancreatitis ranges from 13 to 45 per 100,000 population years, and it is rising worldwide probably due to a combination of risk factors, such as obesity, alcohol abuse, and gallstone disease [1–3]. According to the Revised Atlanta Classification, acute pancreatitis could be classified as mild AP (MAP), moderately severe AP (MSAP), and severe AP (SAP). Although the majority of patients (80%–85%) suffer from MAP with a mortality rate less than 3%, the patients developing severe acute pancreatitis (SAP) have a mortality rate of 13–35% [4]. Numbers of researches have shown that the first 48 hours after symptom onset are critical for effective treatment and good outcome. Thus, a good accurate predictor of the clinical course of AP to identify patients with risk of developing complications or death is essential in AP therapy [5].

HHLA2, also named as B7-H5 or B7-H7, is a newly discovered member of B7 family. Several studies showed that HHLA2 was potentially involved in cancer progression through immune regulation. Upregulation of HHLA2 was associated with poor clinical features and outcomes in breast cancer, clear cell renal cell carcinoma, colorectal cancer, and osteosarcoma, whereas downregulation of HHLA2 might be beneficial for gastric cancer and epithelial ovarian cancer [6, 7]. It can regulate a variety of immune cells, including B cells, T cells, and endothelial cells by binding to its putative receptor(s) on these cells [8].

Although the pathogenesis of AP is still unclear, the role of T lymphocyte–costimulatory pathways has been implicated in the development of this condition [9, 10]. Different studies showed that the members of the B7 family, which function as ligands in costimulatory pathways, had significant clinical significance in AP. Both sB7-H2 and membrane B7-H2 on CD14^+^CD16^+^ cells were specifically increased in AP patients when compared with healthy control, and upregulation of sB7-H2 might be a useful marker in the clinical diagnosis of AP [11, 12]. The level of sB7-H4, another member of the B7 family, was significantly related to the severity and poor prognosis of AP. Besides, upregulation of B7-H3 was also observed in AP patients, and applying anti-B7-H3 monoclonal antibody could ameliorate l-arginine-induced acute pancreatitis in mice via attenuating the inflammatory response [13]. However, according to our knowledge, the role of B7-H5 in AP has not been studied so far.

In this study, we measured the levels of soluble B7-H5 (sB7-H5) in AP patients and analysed the correlation between the levels of sB7-H5 with clinical features and cytokines. We found the levels of sB7-H5 were continually increased in AP patients within 72 hours, and the increasing of sB7-H5 might be a useful diagnostic, severity, and prognosis biomarker for AP patients.

## 2. Materials and Methods

### 2.1. Patients

This retrospective study was approved by the Ethics Committee of First Affiliated Hospital of Soochow University (Ethics Inspection No: 2021 No. 132). 30 MAP patients, 25 MSAP patients, 20 SAP patients, 20 patients with other abdominal pain without AP, and 20 healthy control individuals (recruited from physical examination) were collected from 2019.1 to 2020.12. Oral and written consents were provided from all patients. The patients were diagnosed according to the revised Atlanta classification for AP in 2012. The venous blood samples were collected on admission and after 72 hours. Plasma was separated by centrifugation and stored at -80°C until further analysis.

### 2.2. sB7-H5 and Cytokine Measurement

sB7-H5 and cytokines were detected using ELISA following the Manufacturer's manual. The Human Platelet receptor Gi24 (C10orf54) ELISA kit (CSB-EL002961HU, Cusabio technology, Wuhan, Hubei, China), Human IL-6 ELISA Kit (70-EK106/2-96, MultiSciences Biotech, Hangzhou, Zhejiang, China), Human IL-10 ELISA Kit (70-EK110/2-96, MultiSciences Biotech, Hangzhou, Zhejiang, China), and Human TNF-*α* ELISA Kit (70-EK182-96, MultiSciences Biotech, Hangzhou, Zhejiang, China) were used.

### 2.3. Statistical Analysis

Data are collected and analysed using Graphpad prism (version 8, Graphpad Software, San Diego, CA, USA) and shown as mean ± SD; the difference between 2 groups was evaluated using the Student's *t*-test; differences among multiple groups were evaluated by ANOVA and followed by the least significant difference (LSD) *t*-test. The relationships between sB7-H5 with cytokines were analysed via the Pearson correlation analysis. The receiver operating characteristic (ROC) curves with calculation of the sensitivity and specificity were calculated. A *p* value < 0.05 was considered as statistical significance.

## 3. Results

### 3.1. Levels of sB7-H5 Were Upregulated among AP Patients

**Figure 1:**
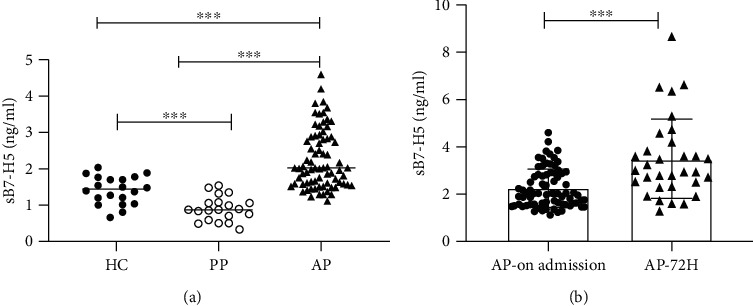
Levels of sB7-H5 were increased in AP patients. (a) Levels of sB7-H5 were significantly increased in AP patients and decreased in abdominal pain patients (PP) when compared with healthy controls (HC). (b) Levels of sB7-H5 of AP patients at 72 hours after admission were significantly increased than those on admission. ^∗∗∗^*p* < 0.001.

To determine the changes of sB7-H5 in AP patients, the healthy group (*n* = 20), abdominal pain without AP group (*n* = 20), and the AP group (*n* = 75) were enrolled. The basic clinical parameters of the AP patients, abdominal pain group, and healthy group are shown in supplement Table 1. There were no significant differences in age and sex among the three groups, and the levels of cytokines such as IL-6, IL-10, and TNF-*α* were significantly upregulated in AP patients, which were in line with previous studies [14]. The levels of sB7-H5 levels in the AP group (2.26 ± 0.81 ng/mL) were significantly higher than those in the healthy group (1.41 ± 0.38 ng/mL), whereas the sB7-H5 levels in the abdominal sepsis group (0.90 ± 0.34 ng/mL) were significantly lower than those in the healthy group (Figure 1(a)). The results indicated that sB7-H5 levels were specifically increased in AP patients. Furthermore, we determined whether the levels of sB7-H5 were changed with time in AP patients. The results showed that the levels of sB7-H5 at 72 hours (3.50 ± 1.69 ng/mL) after admission were still increased than those on admission (Figure 1(b)). These results indicated that the levels of sB7-H5 were upregulated and continuously increasing in AP patients.

### 3.2. Levels of sB7-H5 Function as a Diagnostic Marker

**Figure 2:**
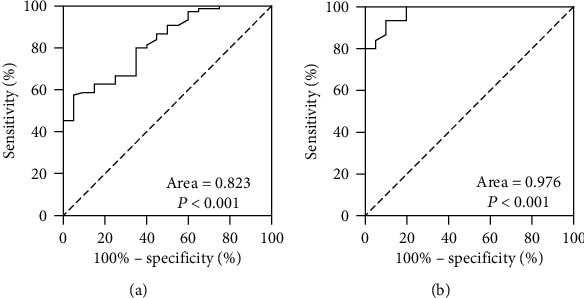
ROC curves of sB7-H5 in differentiating between health with AP (a) and between abdominal pain patients with AP (b).

To assess the value of sB7-H5 in differentiating AP patients among the three groups, ROC curve analyses were used. The areas under the ROC curves (AUCs) were 0.823 and 0.976 in differentiating between the AP with the healthy group and AP with abdominal pain (Figures 2(a) and 2(b)). When both using 1.36 ng per mL as the cut-off value, the sensitivity and specificity of sB7-H5 distinguishing AP from the healthy group and AP from abdominal pain were 78.9% and 86.4%, 93.3%, and 90.0%, respectively. Considering the stable expression of sB7-H5 in AP patients, sB7-H5 is a potential stable AP diagnostic marker with good performance.

### 3.3. Levels of sB7-H5 Were Correlated with the Severity of AP

**Figure 3:**
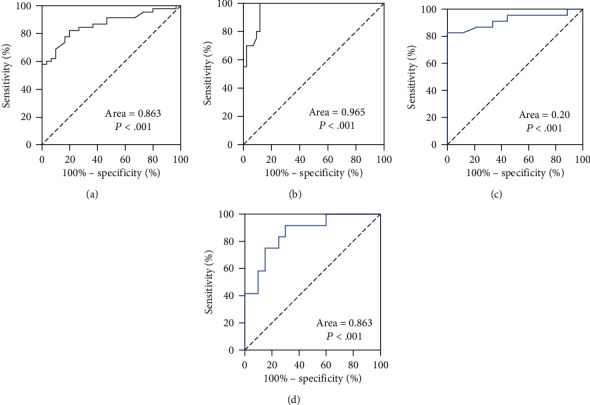
ROC curves of sB7-H5 in predicting the severity of AP. (a) and (b) ROC curves of sB7-H5 on admission in differentiating between MAP + MSAP and SAP (a), MAP and MSAP + SAP (b) ROC curves of sB7-H5 at 72 hours after admission in differentiating between MAP + MSAP and SAP (c), MAP and MSAP + SAP (d).

The stable upregulation of sB7-H5 in AP patients indicated that it specifically responded to the pathological processes of AP. Considering the clinical parameters and cytokines function predictors of severity in AP, we firstly analysed the correlations between sB7-H5 and the inflammatory response in AP patients. As shown in Table 1, there were significant correlations between the serum sB7-H5 expression level and the levels of WBC, GLU, LDH, Ca^2+^, AST, PLT, IL-6, IL-10, TNF-*α*, IFN-*γ*, and scoring systems. The significant correlation between sB7-H5 with clinical parameters and cytokines suggested it could function as a predictor for the severity of AP. To further confirm the value of sB7-H5 in differentiating between MAP, MSAP, and SAP groups, ROC curve analyses were used. The AUCs were 0.863 and 0.972 in differentiating between MAP + MSAP and SAP, MAP, and MSAP + SAP groups on admission, respectively. Using 1.90 ng/mL or 2.538 ng/mL as the cut-off values for distinguishing MAP + MSAP from SAP or MAP from MSAP + SAP, the sensitivity and specificity of sB7-H5 were 82.8% and 80%, 100% and 90.9%, respectively (Figures 3(a) and 3(b)). Moreover, the AUCs of sB7-H5 for differentiating those groups at 72 hours were 0.923 and 0.863, respectively, and the sensitivity and specificity were 82.6%, 100% and 91.7%, 70% (Figures 3(c) and 3(d)). These results indicated that sB7-H5 could identify the severity of AP.

### 3.4. Levels of sB7-H5 Were Correlated with the Prognosis of AP

**Figure 4:**
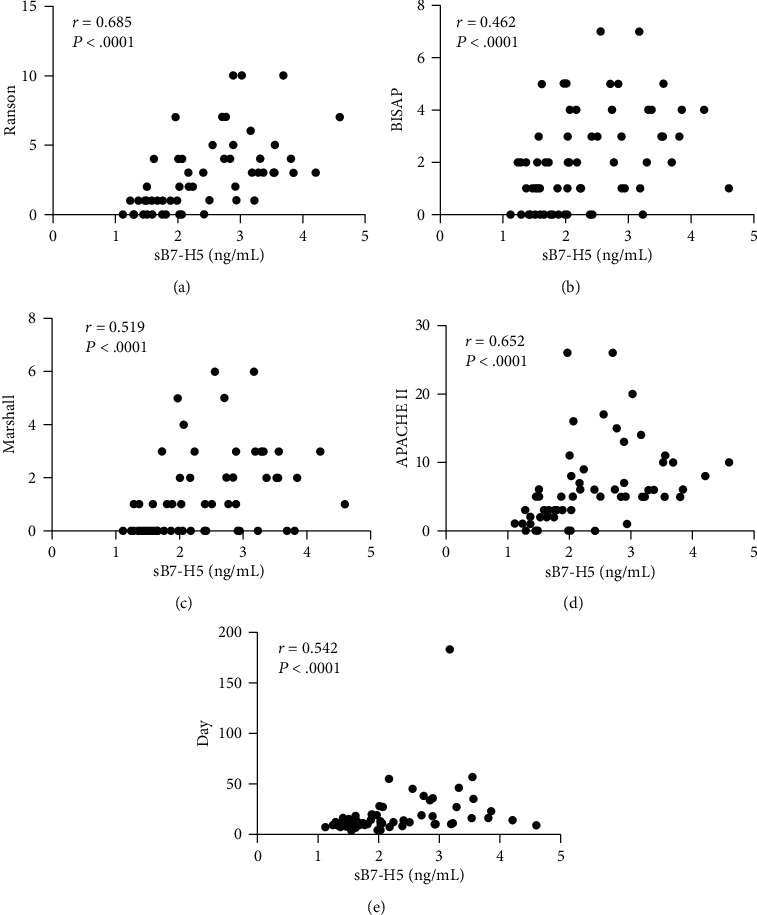
Correlations between the serum sB7-H5 expression level with Ranson score (a), BiSAP score (b), Marshall score (c), APACHE II score (d), and length of stay (e).

**Figure 5:**
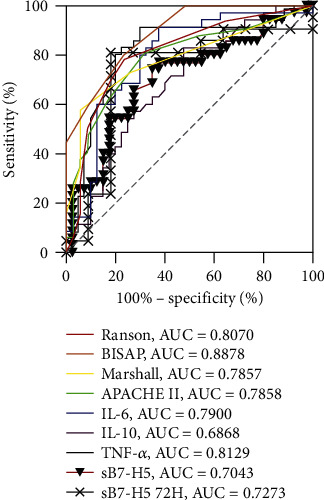
ROC curves of sB7-H5, scoring systems, and cytokines in predicting the outcomes of AP.

To determine the relationship between the levels of sB7-H5 with the prognosis of AP, we first divided the AP patients into two groups according to the median level of sB7-H5 to compare the local complication and length of stay in the two groups. As shown in Table 2, the local complication and length of stay of low levels of the sB7-H5 group were significantly less than those in high levels of the sB7-H5 group. The results indicated the levels of sB7-H5 were related with the outcome of AP. Considering the scoring systems function as an early assessment of the prognosis of acute pancreatitis [5], we further determined the correlation between levels of sB7-H5 with the outcomes of AP patients. Levels of sB7-H5 were significantly correlated with Ranson score (*R* = 0.552, *p* < 0.001), BiSAP score (*R* = 0.512, *p* < 0.001), Marshall score (*R* = 0.462, *p* < 0.001), APACHE II score (*R* = 0.519, *p* < 0.001), and length of stay (*R* = 0.542, *p* < 0.001) (Figures 4(a)–4(e)). Furthermore, the ROCs of sB7-H5, the scoring systems, and cytokines in the prediction of local complications were plotted. The AUCs of sB7-H5 Day1, sB7-H5 Day3, Ranson score, BiSAP score, Marshall score, APACHE II score, IL-6, IL-10, and TNF-*α* were 0.704 (*p* = 0.0024), 0.727 (*p* = 0.0373), 0.796 (*p* < 0.001), 0.888 (*p* < 0.001), 0.786 (*p* < 0.001), 0.751 (*p* < 0.001), 0.790 (*p* < 0.001), 0.687 (*p* = 0.005), and 0.813 (*p* < 0.001) (Figure 5). The results indicated that the levels of sB7-H5 were correlated with the outcomes of AP.

## 4. Discussion

AP is a progressive systemic inflammatory response (SIRS), and the mortality of SAP could be as high as 30% if without timely and appropriate treatment. Early diagnosis of AP and identifying SAP patients is the key point for improved prognosis, but still difficult, thus, there is a need for exploring rapid, long-lasted, and useful biomarkers. Although the pathogenesis of AP is still not fully understood, activation of T cells not only played an important role in the early local as well as systemic immune responses in AP but also directly involved in organ damage, especially in SAP [15]. The severe form of AP is closely related to the extent and systemic immune response [15]. The B7 family is one of the most important families in the costimulatory signal pathways which function as the second signal for T cell activation [16]. Massive studies focused on the immune checkpoint roles for cancer treatment of the B7 family and showed the importance of this family in immune response regulation [7, 17]. Members of the B7 family might also participate in the development of acute pancreatitis [13].

A few studies on members of this family in AP disclosed that sB7-H2, sB7-H4, and PD-L1 (sB7-H1) could function as biomarkers for diagnosis and severity identification. Huang et al. (2015) showed sB7-H2 could distinguish MSAP + SAP from MAP with 77.8% sensitivity and 80.0% specificity, whereas it distinguished SAP from MSAP + MAP with 92.0% sensitivity and 86.0% specificity [11]. Cheng et al. (2020) revealed that sB7-H4 could differentiate between health and AP, MAP and MSAP, and MSAP and SAP groups. The AUC for those groups were 0.958, 0.811, and 0.826, respectively, and the sensitivity and specificity could reach 86.5% and 96%, 81.8% and 67.9%, and 71.8% and 83.3%, respectively [18]. According to Chen et al. (2017), sPD-L1 was an independent risk factor for infectious complications in AP, and the AUC of sPD-L1 for predicting infectious complications was 0.721, which were similar with lymphocyte count [19, 20]. In this study, we evaluated the role of sB7-H5 in AP, and this is the first study of sB7-H5 in AP according to our knowledge.

We first found the levels of sB7-H5 were able to distinguish AP patients from the healthy group or abdominal pain group. Interestingly, the sensitivity and specificity of sB7-H5 to distinguish AP from abdominal pain were better than it for AP from healthy. The contrary trends of sB7-H5 in abdominal pain and acute pancreatitis indicated the different roles of sB7-H5 in these diseases. Second, our results also revealed that levels of sB7-H5 could function as markers of inflammation and identify severe AP patients. Levels of sB7-H5 were positively with the indicators of inflammatory response, including WBC, GLU, LDH, AST, IL-6, IL-10, and TNF-*α*; and further ROC analysis showed the AUC of sB7-H5 to identify the SAP increased from 0.863 on admission to 0.923 at 72 hours. These results indicated sB7-H5 is a stable biomarker for AP severity, and its stability provides a relative long window for detection. Since the outcomes of AP patients are closely related with the severity, we further compared the value of sB7-H5 in prognosis prediction. We also found the AP patients with higher levels of sB7-H5 had significantly more local complication and length of stay. And the AUC of sB7-H5 to predict the local complication increased from 0.704 on admission to 0.727 at 72 hours. These results revealed the prediction value of sB7-H5 in diagnostic, severity, and prognosis of acute pancreatitis.

Considering the clinical features of AP, SAP patients should be identified as soon as possible. Thus, great efforts have been made in the identification of biomarkers (such as acute phase proteins, cytokines, activation peptides of pancreatic proteases, and leukocyte-derived enzymes) that might be used for early risk and severity stratification in AP patients [5, 21]. However, all of these biomarkers and scoring systems have limits. For example, acute phase proteins, such as C-reactive protein (CRP), serum amyloid A protein (SAA), lipopolysaccharide-binding protein (LBP), and pentraxin 3 (PTX 3), are nondisease specific, whereas the scoring systems are unsuitable in clinical practice due to their complex and time-consuming [22–24]. In this study, we found the levels of sB7-H5 could identify SAP with good sensitivity and specificity. Using the levels of sB7-H5 for at 72 hours, the sensitivity and specificity in differentiating between SAP from MAP and MSAP + SAP groups could reach 82.6% and 100%. The high sensitivity indicated that using sB7-H5 for severity prediction could minimize the false negative results, which is important for AP treatment [25].

Since there are few studies about sB7-H5 in AP, the mechanisms of sB7-H5 in AP are still unclear. According to the role of sB7-H5 in various cancers, it may have both coinhibitory functions in inhibiting the proliferation of human CD4 and CD8 T cells and the production of T cell cytokines or costimulatory functions in immune signalling [26]. In our study, the positive relationship between sB7-H5 and cytokines suggested a costimulatory function of sB7-H5 contributing to the progress of inflammation and severity in AP.

## 5. Conclusions

This study reveals that upregulation of sB7-H5 in patients with AP could function as a biomarker for diagnosis, severity, and prognosis. However, this study is limited by the relatively small enrolled number of patients and the lack of patients with organ failure or in-hospital mortality. Further studies with multicentre, large samples may strengthen the value of sB7-H5 in AP diagnostics, severity, and prognosis. Besides, the stable upregulation of sB7-H5 indicates an important role of sB7-H5 in AP, and further studies on the role and mechanism of sB7-H5 may deepen our study on the pathogenesis of AP. Disclose the immune function of sB7-H5 might provide a potential therapy target of AP.

## Figures and Tables

**Table 1:** Correlations between the sB7-H5 expression level with clinical parameters and cytokines.

Clinical parameters/cytokines	Mean ± SD	*r*	*p*
IL-6 (pg/ml)	37.96 ± 22.67	0.722	<0.0001
IL-10 (pg/ml)	34.00 ± 23.58	0.505	<0.0001
TNF-*α* (pg/ml)	1245.77 ± 1005.35	0.685	<0.0001
IFN-*γ* (pg/ml)	41.19 ± 20.71	0.701	<0.0001
WBC (10^/L)	11.47 ± 5.47	0.34	0.004
HCT (L/L)	1.43 ± 6.06	0.067	0.579
PLT (10^9/L)	196.52 ± 77.79	0.244	0.041
PDW (%)	15.45 ± 2.12	0.179	0.16
PCT (%)	2.19 ± 7.25	0.248	0.178
CRP (mg/L)	155.79 ± 141.63	0.202	0.194
Ca^2+^ (mmol/L)	1.91 ± 0.44	-0.323	0.006
AST (U/L)	121.16 ± 275.77	0.306	0.009
ALT (U/L)	112.96 ± 194.72	0.147	0.224
CR (umol/L)	74.69 ± 61.15	0.089	0.462
BUN (mmol/L)	5.84 ± 3.35	0.009	0.942
GLU (mmol/L)	7.53 ± 3.36	0.33	0.008
LDH (U/L)	365.63 ± 490.00	0.516	<0.0001
Alb (g/L)	36.49 ± 5.29	-0.171	0.177
Samy (U/L)	857.94 ± 983.69	0.06	0.629
Lac (mmol/L)	1.73 ± 1.12	-0.053	0.734
RANSON score	2.53 ± 2.58	0.685	<0.0001
MARSHALL score	1.15 ± 1.57	0.519	<0.0001
APACHE score	5.88 ± 5.73	0.652	<0.0001
BISAP score	1.97 ± 1.83	0.462	<0.0001

**Table 2:** Correlations between sB7-H5 levels with clinical characteristics of AP.

Clinical characteristics	Levels of sB7-H5		*t*/*p*
	Low (*n* = 37)	High (*n* = 38)	
Local complication	11	24	8.417/0.004
Hospital stays (days)	1.58 ± 5.07	25.73 ± 31.75	3.626/*p* < 0.0001
RANSON score	1.06 ± 1.48	2.75 ± 1.80	3.939/*p* < 0.0001
BISAP score	1.18 ± 1.49	2.69 ± 1.83	3.632/*p* < 0.0001
Marshall score	0.48 ± 1.06	1.77 ± 1.72	3.792/*p* < 0.0001
APACHE II score	3.26 ± 4.78	6.66 ± 5.39	3.834/*p* < 0.0001
IL-6 (pg/ml)	23.45 ± 11.25	52.09 ± 22.12	5.436/*p* = 0.0002
IL-10 (pg/ml)	22.47 ± 14.48	45.21 ± 25.41	4.101/*p* = 0.0003
TNF-*α* (pg/ml)	571.35 ± 398.80	1902.44 ± 984.38	5.267/*p* = 0.0004

## Data Availability

All experiment methods and the result generated or used during the study appear in the submitted article.

## Supplementary Materials

Supplementary Materials Supplement Table 1: basic clinical parameters of the AP patients, abdominal pain without AP group and healthy group.
