# Supplementary material for: Soluble B7-H5 Is a Novel Diagnostic, Severity, and Prognosis Marker in Acute Pancreatitis
Source: Biomed Res Int. 2021 Oct 8;2021:1223850. doi: 10.1155/2021/1223850 (PMC8519671; doi:10.1155/2021/1223850)
Supplement: Supplementary Materials — Supplement Table 1: basic clinical parameters of the AP patients, abdominal pain without AP group and healthy group. [file 1223850.f1.zip › Supplement table1 Basic clinical parameters of the AP patients.docx]

**Supplement table1 Basic clinical parameters of the AP patients, abdominal pain without AP group and healthy group.**

| Characteristics | healthy(n=20) | abdominal pain(n=20) | AP(n=75) | ***p*** |
| --- | --- | --- | --- | --- |
| Male/female | 10/10 | 11/9 | 49/26 | 0.386 |
| Age(years) | 42.95±10.25 | 50.10±18.00 | 49.44±15.86 | 0.326 |
| IL-6（pg/ml） | 0.41±0.26 | 9.72±4.22 | 37.96±22.67 | <0.001 |
| IL-10（pg/ml） | 11.73±8.18 | 16.59±14.68 | 33.99±23.58 | <0.001 |
| TNF-α（pg/ml） | 74.71±24.14 | 240.42±68.26 | 1245.77±1005.35 | <0.001 |
| IFN-γ（pg/ml） | 30.13±10.65 | 38.77±10.10 | 41.19±20.71 | 0.063 |
