# Supplementary material for: Soluble B7-H5 Is a Novel Diagnostic, Severity, and Prognosis Marker in Acute Pancreatitis
Source: Biomed Res Int. 2021 Oct 8;2021:1223850. doi: 10.1155/2021/1223850 (PMC8519671; doi:10.1155/2021/1223850)
Supplement: Supplementary Materials — Supplement Table 1: basic clinical parameters of the AP patients, abdominal pain without AP group and healthy group. [file 1223850.f1.zip › Supplementary Description.docx]

## Supplementary Description

**Supplement table1 Basic clinical parameters of the AP patients, abdominal pain without AP group and healthy group.**
